# Supplementary material for: Ketogenic Diet Affects Sleep Architecture in C57BL/6J Wild Type and Fragile X Mice
Source: Int J Mol Sci. 2023 Sep 22;24(19):14460. doi: 10.3390/ijms241914460 (PMC10572443; doi:10.3390/ijms241914460)
Supplement: Supplementary file 1 [file ijms-24-14460-s001.zip › ijms-2575743-supplementary.pdf]

Supplementary Materials

Ketogenic Diet Affects Sleep Architecture in C57BL/6J Wild Type and Fragile X Mice

Pamela R. Westmark [1], Aaron K. Gholston [1,2], Timothy J. Swietlik [1], Rama K. Maganti [1] and Cara J. Westmark [1,2,*]

[1] Department of Neurology, University of Wisconsin, Madison, WI 53706, USA; prwestmark@wisc.edu (P.R.W.); agholston@wisc.edu (A.K.G.); tswietlik@wisc.edu (T.J.S.); maganti@neurology.wisc.edu (R.K.M.)
[2] Molecular Environmental Toxicology Center, University of Wisconsin, Madison WI 53706, USA
* Correspondence: westmark@wisc.edu; Tel.: +1-608-262-9730

A

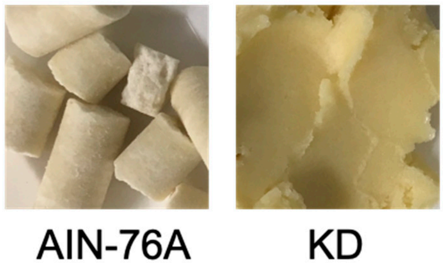

B

Diet Composition

Component	AIN-76A	KD
Protein	18.1%	8.6%
Fat	5.1%	75.1%
Fiber	4.8%	4.8%
Ash	2.9%	3.0%
Carbohydrate	65.2%	3.2%
Energy Density	3.79kcal/g	7.24kcal/g

C

Diet Components

AIN-76A	KD
sucrose	lard
casein	butter
corn starch	corn oil
corn oil	casein
cellulose	cellulose
mineral mix	mineral mix
vitamin mix	vitamin mix
DL-methionine	dextrose
choline bitartrate	

Figure S1. Diet Composition. (**A**) Test diets included control AIN-76A (Bio-Serv, catalog number F1515, Flemington, NJ, USA) and KD (Bio-Serv, catalog number F3666). (**B**) AIN-76A is a purified ingredient diet with a nutritional profile of 18.1% protein, 5.1% fat, 4.8% fiber, 2.9% ash, 65.2% carbohydrate, and an energy density of 3.79 kcal/g. The KD is a modified AIN-76A high fat paste with a nutritional profile of 8.6% protein, 75.1% fat, 4.8% fiber, 3.0% ash, 3.2% carbohydrate, and an energy density of 7.24 kcal/g. The ratio of fat to carbohydrate and protein in the KD is 6:1. (**C**) Ingredients for AIN-76A are sucrose, casein, corn starch, corn oil, cellulose, mineral mix, vitamin mix, DL-methionine, and choline bitartrate. The ingredients for KD are lard, butter, corn oil, casein, cellulose, mineral mix, vitamin mix, and dextrose.

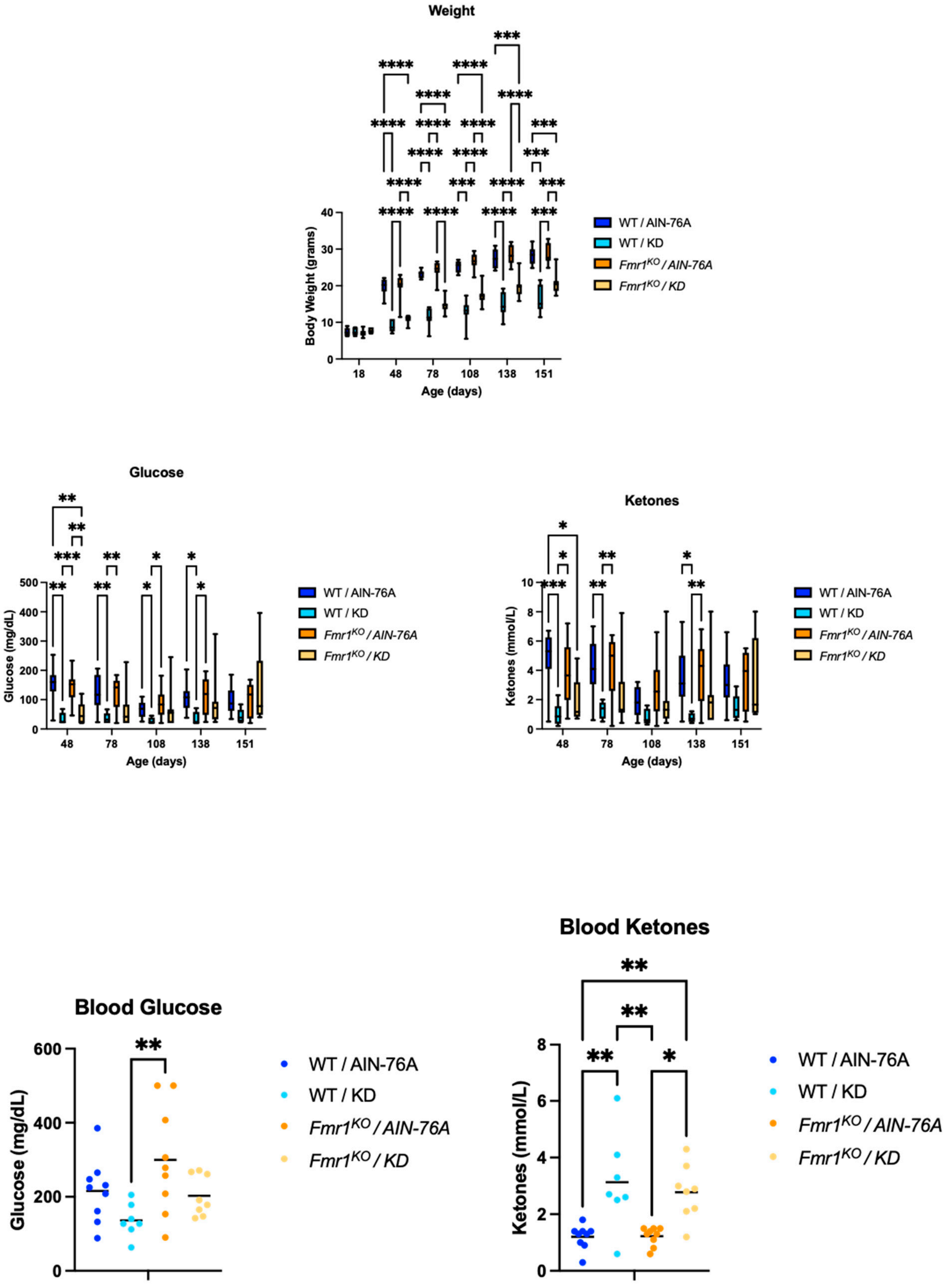

Figure S2. Biometrics as a Function of KD. Mice were weighed at P18 before KD and 30, 60, 90 and 120 days post commencement of KD as well as prior to EEG/EMG surgery. Urine glucose and ketone measurements were assessed at the same time points post-KD treatment. Blood glucose and ketone measurements were assessed at euthanization. Statistics with GraphPad Prism included 1- and 2-way ANOVA with post-hoc Tukey multiple comparison tests. Asterisks indicate statistical significance where * $p < 0.05$, ** $p < 0.01$, *** $p < 0.001$, **** $p < 0.0001$.

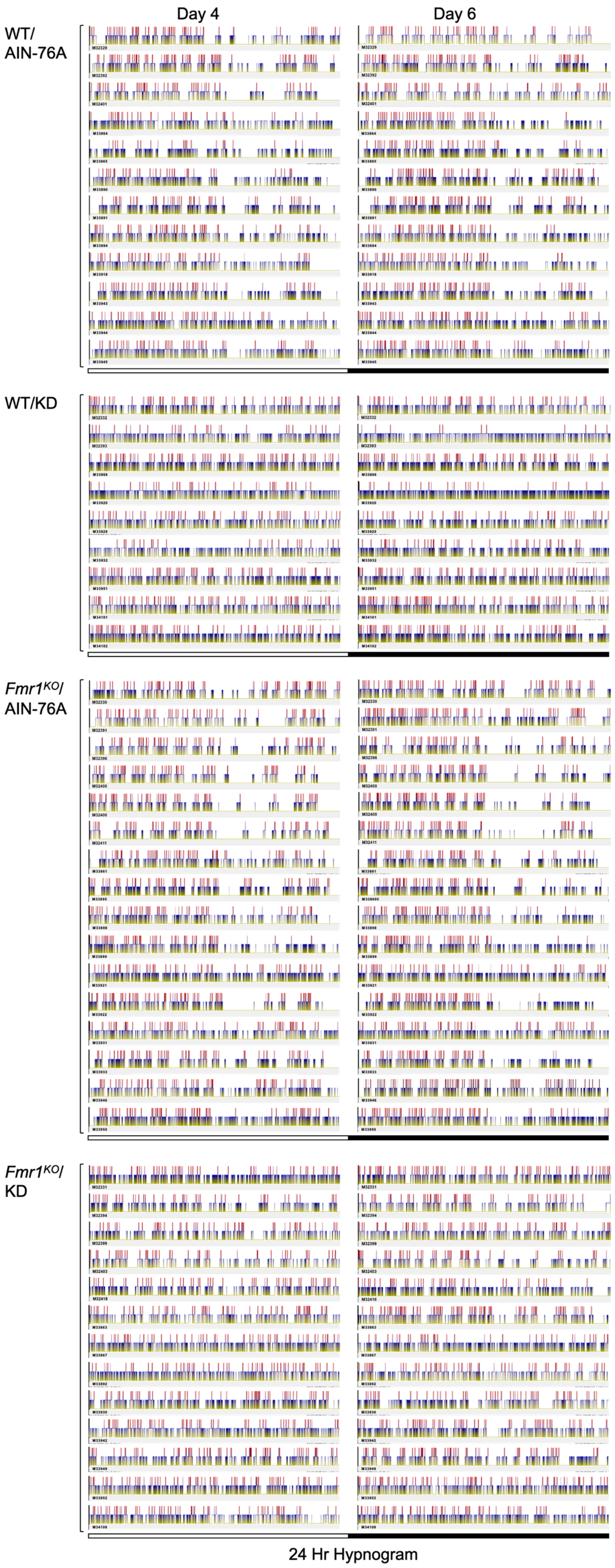

Figure S3. Hypnograms of 24-Hour Sleep-Wake Cycles as a Function of Genotype and Diet. WT and *Fmr1*^{KO} male mice maintained on a 12-hour lights on/12-lights off cycle were fed AIN-76A versus

KD. Hypnograms, which represent sleep stages as a function of time, were generated with Sirenia Sleep® software after manual scoring of sleep states of the EEG/EMG recordings. Mice included WT/AIN-76A (n = 12), WT/KD (n = 9), *Fmr1*^{KO}/AIN-76A (n = 16), and *Fmr1*^{KO}/KD (n = 13). The red signal denotes REM sleep, blue signal denotes NREM sleep, and yellow signal denotes wake state.

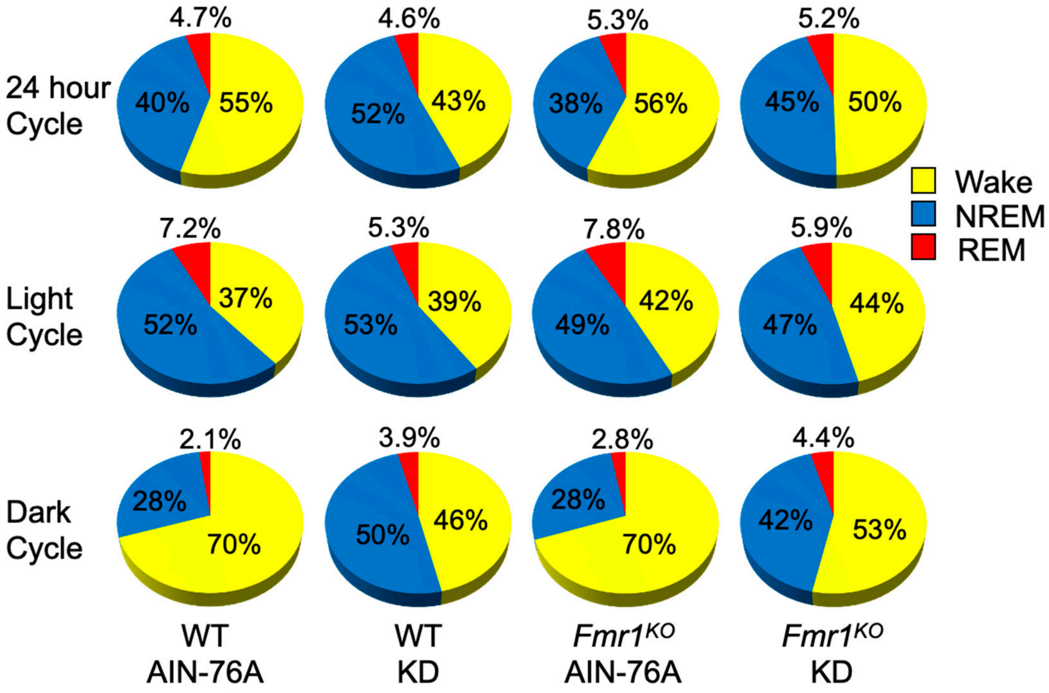

Figure S4. Pie Charts of 24-Hour Sleep-Wake Cycles as a Function of Genotype and Diet. WT and *Fmr1*^{KO} male mice maintained on a 12-hour lights on / 12-lights off cycle were fed AIN-76A versus KD. Sleep stage data were plotted in pie charts. Data were averaged from WT/AIN-76A (n = 12), WT/KD (n = 9), *Fmr1*^{KO}/AIN-76A (n = 16), and *Fmr1*^{KO}/KD (n = 13) mice. Yellow denotes wake state, blue denotes NREM sleep, and red denotes REM sleep. Some pie charts do not achieve 100% due to artifact sections in the EEG recordings that could not be scored (for example, mouse bumps head on water bottle nozzle or electrical interference).

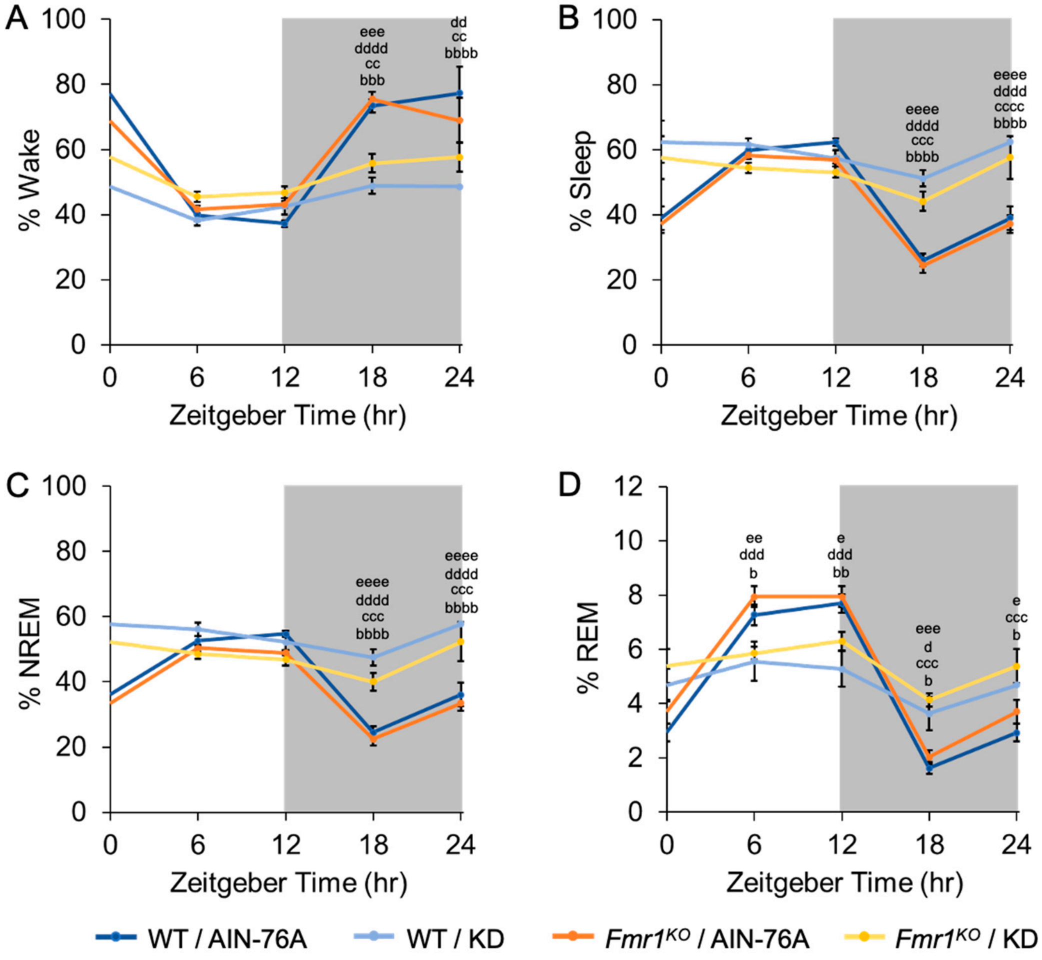

Figure S5. Effect of KD on Sleep Architecture in WT and *Fmr1*^{KO} Mice as a Function of Time of Day. EEG/EMG recordings were scored for wake, sleep, NREM and REM activity and the data parsed into 6-hour bins starting at Zeitgeber time zero. The average percent sleep state was plotted versus Zeitgeber time bin for (**A**) % wake, (**B**) % sleep, (**C**) % NREM, and (**D**) % REM. Mouse cohorts included WT fed AIN-76A (n = 12), WT fed KD (n = 9), *Fmr1*^{KO} fed AIN-76A (n = 16), and *Fmr1*^{KO} fed KD (n = 13). Statistics with GraphPad Prism included 2-way ANOVA with repeated measures with post-hoc Tukey multiple comparison tests. Key for statistical significance: WT/AIN-76A versus *Fmr1*^{KO}/AIN-76A = "a", WT/AIN-76A versus WT/KD = "b", WT/AIN-76A versus KO/KD= "c", WT/KD versus KO/AIN-76A= "d", KO/AIN-76A versus *Fmr1*^{KO}/KD= "e", and WT/KD versus *Fmr1*^{KO}/KD= "f"; and x p<0.05, xx p<0.01, xxx p<0.001, xxxx p<0.0001 where x= a, b, c, d, e, or f.

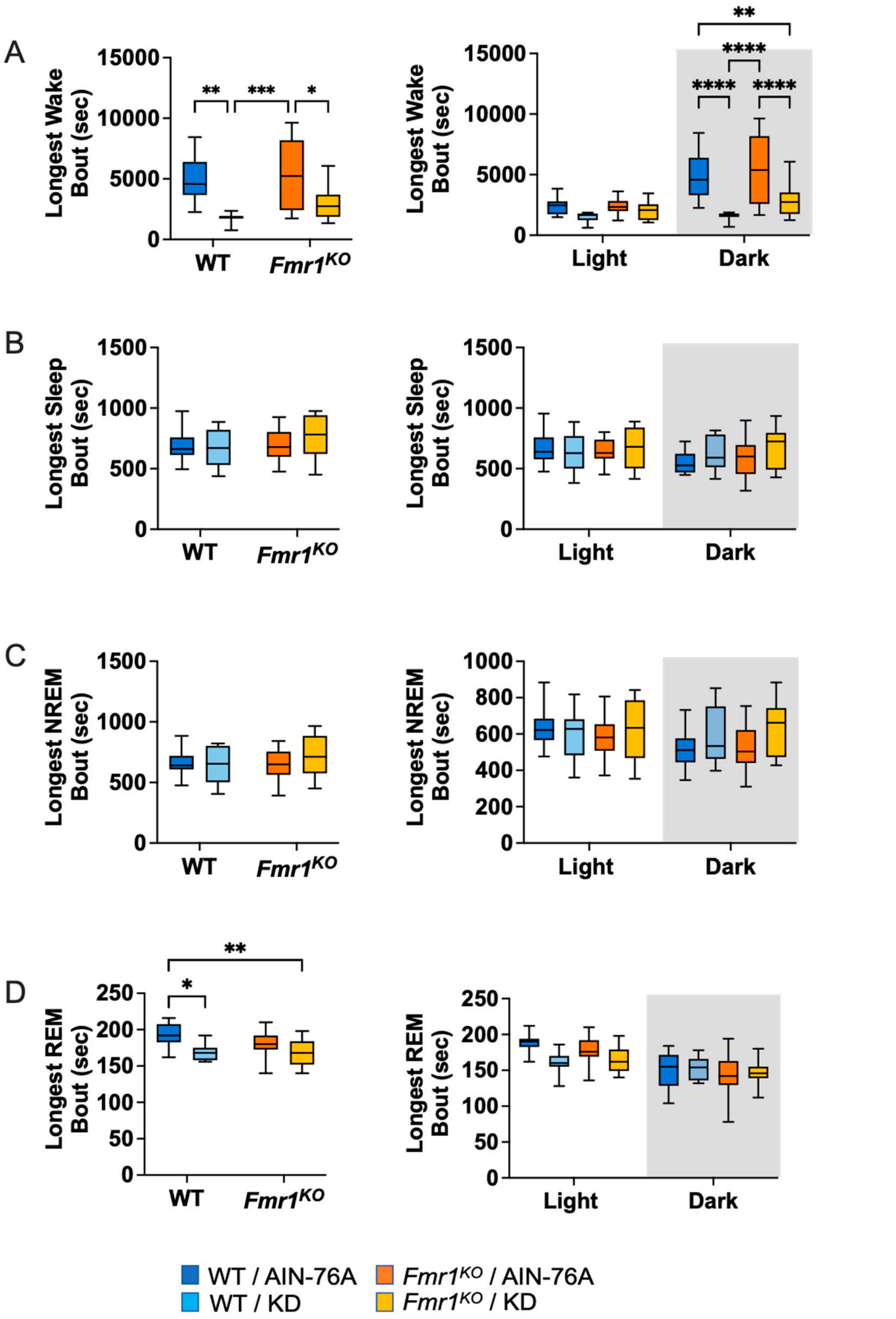

Figure S6. Effect of KD on Additional Sleep Micro-Architecture Phenotypes in WT and *Fmr1*^{KO} Mice as a Function of Light and Dark Cycles. EEG/EMG recordings were scored for wake, NREM and REM activity and the data parsed into 24-hour full day and 12-hour light/dark bins starting at Zeitgeber time zero. Phenotype averages were plotted versus bin for (**A**) longest wake bout, (**B**) longest sleep bout, (**C**) longest NREM bout, and (**D**) longest REM bout. Mouse cohorts included WT fed AIN-76A (n = 12), WT fed KD (n = 9), *Fmr1*^{KO} fed AIN-76A (n = 16), and *Fmr1*^{KO} fed KD (n = 13). Statistics with GraphPad Prism included 2-way ANOVA with post-hoc Tukey multiple comparison tests. Key for statistical significance: * $p < 0.05$, ** $p < 0.01$, *** $p < 0.001$, **** $p<0.0001$.

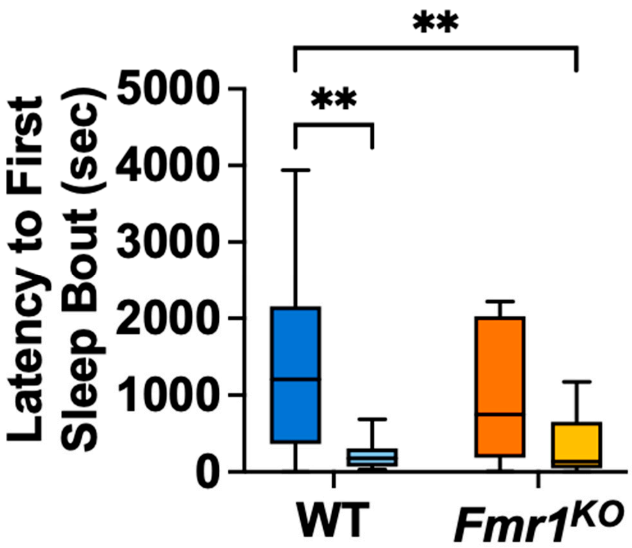

Figure S7. Effect of KD on Latency Time to Enter First Sleep Bout in WT and *Fmr1*^{KO} Mice. EEG/EMG recordings were scored for the latency time to the first sleep bout and the data parsed into 24-hour full day and 12-hour light/dark bins starting at Zeitgeber time zero. The average percent latency time was plotted versus Zeitgeber time bin. Statistics with GraphPad Prism included 2-way ANOVA with post-hoc Tukey multiple comparison tests. Key for statistical significance: ** $p < 0.01$.

Reagents/Materials	Catalog #	Manufacturer/Supplier	City/State
**Reagents:**			
Acetone, ACS Reagent, ≥99.5%	320110-1L	Sigma-Aldrich	St. Louis, MO, USA
Betadine Surgical Scrub Providone-iodine, 7.5%	NDC 67618-151-16	Avrio Health, LP	Stamford, CT, USA
Bupivacaine Hydrochloride Injection, USP,0.75%, 7.5 mg/mL	AX11559	Hospira, Inc.	Lake Forest, IL, USA
Clean Air canisiter	5000	DEE Veterinary Products	Miami Gardens, FL, USA
Hydrogen Peroxide Topical Solution USP	NDC 0869-0871-43	Swan	Smyrna, TN, USA
Isoflurane, USP	NDC 14043-704-06	Patterson Veterinary	Greeley, CO, USA
Meloxican Solution for Injection (5 mg/mL)	ANADA 200-540	Putney, Inc.	Portland, ME, USA
Other general chemicals	----	Fisher Scientific	Waltham, MA, USA
Oxygen USP Medical Pure E CGA #70	OX USPE	Airgas, Inc	Radnor, PA, USA
Puralube Vet Ointment-petroleum ophthamiceye ointment	NDC 17033-211-38	Dechra Veterinary Products	Overland Park, KS, USA
Sodium Chloride Injection, 0.9%, USPsterile	NDC 0409-4888-10	Hospira	Lake Forest, IL, USA
Teets "Cold Cure" Denture Material - Methyl Methacrylate - Coral	----	Co-Oral-Ite Dental Manufacturing Co	Diamond Springs, CA, USA
Teets Denture Material Crosslinking Methyl Methacrylate Liquid Compound	----	Co-Oral-Ite Dental Manufacturing Co	Diamond Springs, CA, USA
Control AIN-76A Diet	F1515	Bio-Serv	Flemington, NJ, USA
Ketogenic Diet AIN-76A-Modified, High Fat, Paste Diet	F3666	Bio-Serv	Flemington, NJ, USA
**Materials:**			
BD 18G1 1/2 Precision Glide Needlegauge needle	305196	Becton Dickenson & Co	Franklin Lakes, NJ, USA
Connector RCPT Socket 100PIN DL. 100 L. 143	ED1316-100-ND/803-43-100-62-001000	Mill-Max Mfc Corp./Digi-Key Electronics	Thief River Falls, MN, USA
Connector Header Through Hole 68 position 0.100"	ED 1312-38-ND/802-10-038-62-001000	Mill-Max Mfc Corp./Digi-Key Electronics	Thief River Falls, MN, USA
Ethilon Nylon Suture Black Monofilament FS-1	699H	Ethicon, LLC	Bridgewater, NJ, USA
Far Infrared Warming Pad	RT-0515	Kent Scintific Corp.	Torrington, CT, USA
KOPF Stereotaxic Alignment Instruments		David KOPF Instruments	Tujunga, CA, USA
Ohmeda Isotec 3 IsoFlurane Anesthetic Vaporizer	Isotec 3	Ohmeda Medical Inc/GE Helathcare	Laurel, MD, USA
PFA Coated Stainless Steel Wire (skull cap-brain)	791400	A-M Systems	Sequim, WA, USA
Puritan Sterile Cotton Tipped 6" Applicators	25-806 1WC	Puritan Medical Products	Guilford, ME, USA
SAC305 Lead-Free Solder (96.3% Sn, 0.7% Cu, 3% Ag)	4900-18G	MG Chemicals	Surrey, British Columbia, Canada
Stainless Steel Screws/electrodes (0.12" diameter)			
Stranded Stainless Steel Wire (skull cap-musculature)	AS 636	Cooner Wire	Chatsworth, CA, USA
WAHL Micro Groomsman Battery Trimmer	Model 5640-600	WAHL Clipper Corp	Sterling, IL, USA
XLTEK EMU40 EEG System	EMU40	Natus Medical, Inc.	Pleasanton, CA, USA
**Software:**			
EDF Browser v2.04	----	Tenius van Beelen	www.teuniz.net/edfbrowser/index.html
GraphPad Prism version 10.0.0 (153)	----	GrapgPad Software, LLC	San Diego, CA, USA
Microsoft 365 Excel™ (version 2306)	----	Microsoft Corporation	Redmond, WA, USA
Sirenia Sleep Pro v1.3.2	----	Pinnacle technology, Inc	Lawrence, KS, USA
Natus Neuro Works EEG Software/xltek	----	xltek-Natus Medical, Inc.	Pleasanton, CA, USA

Figure S8. Reagents and materials.

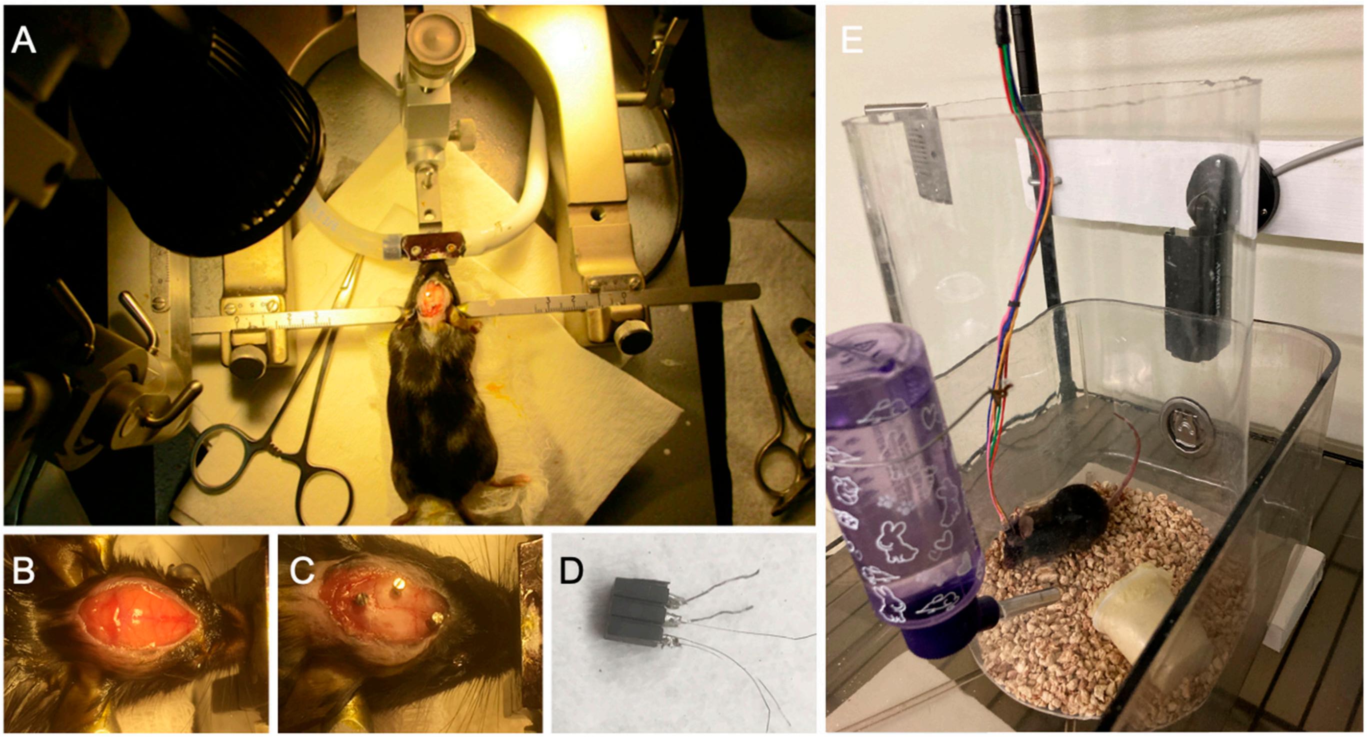

Figure S9. EEG/EMG Electrode Implantation Surgery and Recording. (**A**) The mouse was placed in a stereotaxic frame and held immobile with ear bars and nose clamp. The nose was fixed by placing the teeth of the mouse into the tooth holder of the nose clamp, which also supplies anesthesia and oxygen to the mouse. Eye gel was applied to eyes to protect them from drying out during the surgery. (**B**) A midline incision was made through the skin covering the skull. The periosteum was removed with cotton tipped applicators dipped in 70% alcohol followed by hydrogen peroxide. (**C**) Three holes were drilled in the skull using an 18G needle. Then three stainless steel screws were placed as electrodes with two screws over the frontal (Bregma +1.5 mm and +1 mm laterally) and parietal cortex (Bregma -3 mm and -1 mm laterally) and one screw as the occipital reference (lambda -1 mm at midline). (**D**) Three stainless steel wires from the cap assembly were wrapped around the screws. Two stranded stainless steel wire electrodes were inserted in the nuchal muscles directly behind the skull parallel with the shoulders for EMG recording. (**E**) Mice were allowed to recover from surgery for 3 days and then connected to an EEG recorder via a wire tether and placed in a cylindrical apparatus with *ad libitum* access to water and food during the EEG/EMG recordings.

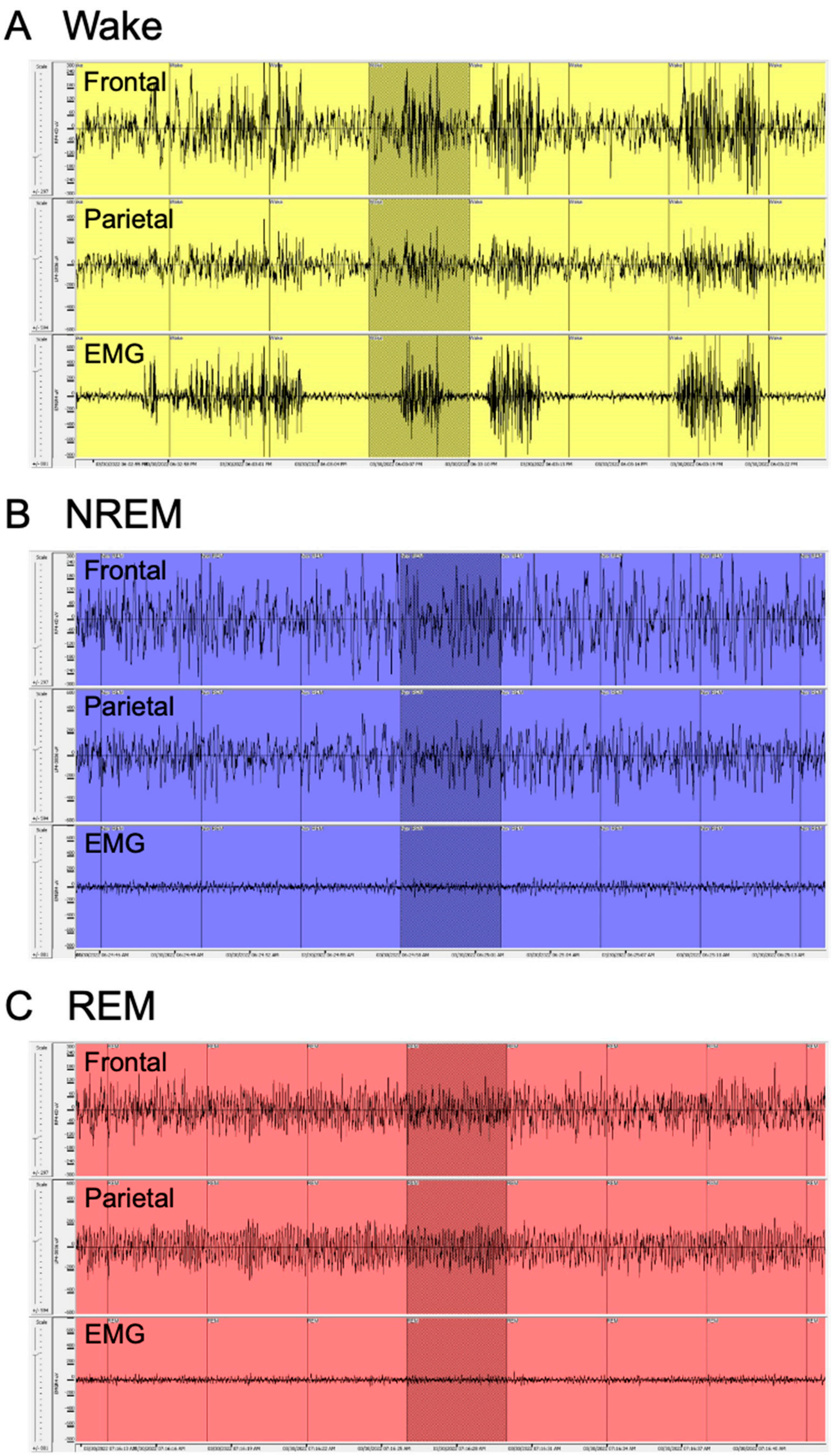

Figure S10. Examples of EEG/EMG Signals. EEG/EMG recordings were manually scored in four second epochs for wakefulness, NREM sleep and REM sleep. (**A**) During wake, the EEG signal is composed of mixed frequencies with low amplitude theta, alpha and beta range activity. The frontal lead exhibits low, regular tone while the parietal lead is rhythmic. The EMG signal indicates high

amplitude activity. When the animal is resting but awake the EMG has a low tone, but no spindles are present in the frontal lead. (**B**) During NREM sleep there is high amplitude, low frequency EEG oscillations (1-4 Hz) with sleep spindles in the frontal and/or parietal electrodes. The EMG muscle tone is low or flat. (**C**) REM sleep has low amplitude theta activity (6–9 Hz) where both the frontal and parietal leads are very rhythmic. There is very low to no EMG tone except for rhythmic heart-rate artifacts.

Table S1. ANOVA Statistics.

Figure 2

% Wake 24-hour bin

Interaction, F (1, 46) = 2.067, P=0.1573

Genotype, F (1, 46) = 6.555, P=0.0138

Diet, F (1, 46) = 33.32, P<0.0001

% Wake 12-hour bins

Time x Cohort, F (3, 46) = 37.23, P<0.0001

Time, F (1, 46) = 325.5, P<0.0001

Cohort, F (3, 46) = 11.96, P<0.0001

Subject, F (46, 46) = 2.287, P=0.0030

% Sleep 24-hour bin

Interaction, F (1, 46) = 2.364, P=0.1310

Genotype, F (1, 46) = 6.167, P=0.0167

Diet, F (1, 46) = 35.06, P<0.0001

% Sleep 12-hour bins

Time x Cohort, F (3, 46) = 26.44, P<0.0001

Time, F (1, 46) = 172.0, P<0.0001

Cohort, F (3, 46) = 9.761, P<0.0001

Subject, F (46, 46) = 1.824, P=0.0021

% NREM 24-hour bin

Interaction, F (1, 46) = 2.308, P=0.1135

Genotype, F (1, 46) = 8.162, P=0.0064

Diet, F (1, 46) = 35.38, P<0.0001

% NREM 12-hour bins

Time x Cohort, F (3, 46) = 31.31, P<0.0001

Time, F (1, 46) = 183.1, P<0.0001

Cohort, F (3, 46) = 11.98, P<0.0001

Subject, F (46, 46) = 2.723, P=0.0005

% REM 24-hour bin

Interaction, F (1, 46) = 0.003487, P=0.9532

Genotype, F (1, 46) = 5.423, P=0.0243

Diet, F (1, 46) = 0.1379, P=0.7121

% REM 12-hour bins

Time x Cohort, F (3, 46) = 21.20, P<0.0001

Time, F (1, 46) = 202.8, P<0.0001

Cohort, F (3, 46) = 2.008, P=10.1260

Subject, F (46, 46) = 1.333, P=0.1667

Figure 3

% Wake 2-hour bins

Time x Genotype/Diet, F (33, 506) = 12.19, P<0.0001

Time, F (11, 506) = 79.05, P<0.0001

Genotype/Diet, F (3, 46) = 11.61, P<0.0001

Subject, F (46, 506) = 5.227, P<0.0001

% Sleep 2-hour bins

Time x Genotype/Diet, F (33, 506) = 10.46, P<0.0001

Time, F (11, 506) = 67.65, P<0.0001

Genotype/Diet, F (3, 46) = 12.73, P<0.0001

Subject, F (46, 506) = 4.604, P<0.0001

% NREM 2-hour bins

Time x Genotype/Diet, F (33, 506) = 12.08, P<0.0001

Time, F (11, 506) = 72.47, P<0.0001

Genotype/Diet, F (3, 46) = 12.50, P<0.0001

Subject, F (46, 506) = 7.183, P<0.0001

<u>% REM 2-hour bins</u>

Time X Genotype/Diet, F (33, 506) = 7.222, P<0.0001

Time, F (11, 506) = 61.88, P<0.0001

Genotype/Diet, F (3, 46) = 2.043, P=0.1209

Subject, F (46, 506) = 3.174, P<0.0001

Figure 4

Wake Bouts 24-hour bin

Interaction, F (1, 46) = 4.436, P=0.0407

Genotype, F (1, 46) = 4.094, P=0.0489

Diet, F (1, 46) = 11.90, P=0.0012

Wake Bouts 12-hour bins

Time x Cohort, F (3, 46) = 3.512, P=0.0224

Time, F (1, 46) = 73.19, P<0.0001

Cohort, F (3, 46) = 5.985, P=0.0016

Subject, F (46, 46) = 4.439, P<0.0001

Sleep Bouts 24-hour bin

Interaction, F (1, 46) = 4.959, P=0.0309

Genotype, F (1, 46) = 3.466, P=0.0691

Diet, F (1, 46) = 13.27, P=0.0007

Sleep Bouts 12-hour bins

Time x Cohort, F (3, 46) = 3.568, P=0.0210

Time, F (1, 46) = 74.19, P<0.0001

Cohort, F (3, 46) = 6.364, P=0.0011

Subject, F (46, 46) = 4.418, P<0.0001

NREM Bouts 24-hour bin

Interaction, F (1, 46) = 4.687, P=0.0356

Genotype, F (1, 46) = 3.556, P=0.0657

Diet, F (1, 46) = 13.05, P=0.0007

NREM Bouts 12-hour bins

Time x Cohort, F (3, 46) = 3.867, P=0.0151

Time, F (1, 46) = 79.88, P<0.0001

Cohort, F (3, 46) = 6.272, P=0.0012

Subject, F (46, 46) = 4.369, P<0.0001

REM Bouts 24-hour bin

Interaction, F (1, 46) = 0.4167, P=0.5218

Genotype, F (1, 46) = 4.235, P=0.453

Diet, F (1, 46) = 0.2075, P=0.6508

REM Bouts 12-hour bins

Time x Cohort, F (3, 46) = 12.35, P<0.0001

Time, F (1, 46) = 153.1, P<0.0001

Cohort, F (3, 46) = 2.263, P=0.0937

Subject, F (46, 46) = 1.154, P=0.3146

Figure 5

Wake Bouts 24-hour bin

Interaction, F (1, 46) = 3.538, P=0.663

Genotype, F (1, 46) = 4.700, P=0.0354

Diet, F (1, 46) = 15.60, P=0.0003

Wake Bouts 12-hour bins

Time x Cohort, F (3, 46) = 12.40, P<0.0001

Time, F (1, 46) = 101.0, P<0.0001

Cohort, F (3, 46) = 7.346, P=0.0004

Subject, F (46, 46) = 2.266, P=0.0032

Sleep Bouts 24-hour bin

Interaction, F (1, 46) = 2.316, P=0.1349

Sleep Bouts 12-hour bins

Time x Cohort, F (3, 46) = 11.18, P<0.0001

Genotype, F (1, 46) = 0.08638, P=0.7702

Diet, F (1, 46) = 0.2842, P=0.5965

Time, F (1, 46) = 30.54, P<0.0001

Cohort, F (3, 46) = 0.7991, P=0.5007

Subject, F (46, 46) = 3.262, P<0.0001

NREM Bouts 24-hour bin

Interaction, F (1, 46) = 1.699, P=0.1989

Genotype, F (1, 46) = 0.02202, P=0.8827

Diet, F (1, 46) = 5.559e-006, P=0.9981

NREM Bouts 12-hour bins

Time x Cohort, F (3, 46) = 8.720, P=0.0001

Time, F (1, 46) = 17.75, P=0.0001

Cohort, F (3, 46) = 0.7404, P=0.5334

Subject, F (46, 46) = 3.342, <0.0001

REM Bouts 24-hour bin

Interaction, F (1, 46) = 0.8284, P=0.3675

Genotype, F (1, 46) = 2.310, P=0.1354

Diet, F (1, 46) = 1.714, P=0.1970

REM Bouts 12-hour bins

Time x Cohort, F (3, 46) = 1.674, P=0.1858

Time, F (1, 46) = 0.5279, P=0.4712

Cohort, F (3, 46) = 1.227, P=0.3106

Subject, F (46, 46) = 1.968, P=0.0118

Figure 6

Time, F (1, 46) = 2.323, P=0.1341

Genotype / Diet, F (3, 46) = 1.562, P=0.2113

Time x Genotype / Diet, F (3, 46) = 4.281, P=0.0095

Subject, F (46, 46) = 3.511, P<0.0001

Supplementary Figure S2

Body Weight

Time, F (2.130, 62.21) = 556.5, P<0.0001

Genotype / Diet, F (3, 30) = 54.39, P<0.0001

Time x Genotype / Diet, F (15, 146) = 26.33, P<0.0001

Urine Glucose

Time, F (2.230, 59.66) = 2.253, P= 0.1083

Genotype / Diet, F (3, 30) = 5.223, P= 0.0051

Time x Genotype / Diet, F (12, 107) = 2.159, P= 0.0188

Urine Ketones

Time, F (2.765, 75.35) = 3.075, P= 0.0363

Genotype / Diet, F (3, 30) = 5.775, P= 0.0031

Time x Genotype / Diet, F (12, 109) = 1.339, P= 0.2073

Blood Glucose

Treatment, F (3, 29) = 4.056, P=0.0160

Blood Ketones

Treatment, F (3, 29) = 9.315, P=0.0002

Supplementary Figure S7

% Wake 2-hour bins

Time x Genotype/Diet, F (9, 138) = 5.399, P<0.0001

Time, F (3, 138) = 47.00, P<0.0001

Genotype/Diet, F (3, 46) = 6.782, P=0.0007

Subject, F (46, 138) = 1.450, P=0.0521

% Sleep 2-hour bins

Time x Genotype/Diet, F (9, 138) = 10.20, P<0.0001

Time, F (3, 138) = 59.51, P<0.0001

Genotype/Diet, F (3, 46) = 10.34, P<0.0001

Subject, F (46, 138) = 2.023, P=0.0009

% NREM 2-hour bins

Time x Genotype/Diet, F (9, 138) = 9.038, P<0.0001

Time, F (3, 138) = 49.26, P<0.0001

Genotype/Diet, F (3, 46) = 11.10, P<0.0001

Subject, F (46, 138) = 2.450, P<0.0001

% REM 2-hour bins

Time x Genotype/Diet, F (9, 138) = 12.19, P<0.0001

Time, F (3, 138) = 106.4, P<0.0001

Genotype/Diet, F (3, 46) = 1.470, P=0.2350

Subject, F (46, 138) = 2.162, P=0.0003
